# Epidemiological characteristics of cancers in patients with end-stage kidney disease: a Korean nationwide study

**DOI:** 10.1038/s41598-021-83164-6

**Published:** 2021-02-16

**Authors:** Min-Jeong Lee, Eunyoung Lee, Bumhee Park, Inwhee Park

**Affiliations:** 1 Department of Nephrology, Ajou University School of Medicine, Suwon, Republic of Korea; 2 Department of Biomedical Informatics, Ajou University School of Medicine, Suwon, Republic of Korea; 3 Office of Biostatistics, Medical Research Collaborating Center, Ajou Research Institute for Innovative Medicine, Ajou University Medical Center, Suwon, Republic of Korea; 4 Department of Medical Sciences, Biomedical Informatics, Graduate School of Ajou University, Suwon, Republic of Korea

**Keywords:** Medical research, Nephrology

## Abstract

Patients with end-stage kidney disease (ESKD) have been reported to have an increased risk of cancer. However, the epidemiological characteristics of cancer in ESKD patients remain unclear. Therefore, this study aimed to investigate the epidemiological characteristics of cancer in ESKD patients and the differences based on the renal replacement therapy provided. Data on ESKD patients were obtained from the South Korean nationwide cohort Health Insurance Review and Assessment Service database. This study included 58,831 eligible patients of the total 813,907 patients diagnosed with ESKD between January 1, 2007 and December 31, 2017. Of the 58,831 ESKD patients, 3292 (5.6%) were newly diagnosed with cancer. The average duration between the diagnosis of ESKD and cancer was 3.3 ± 1.9 years (mean ± standard deviation), with no differences between hemodialysis, peritoneal dialysis, and kidney transplant groups. The most commonly observed cancer sites in ESKD patients were the colorectum, lung, and liver. The incidence of cancer increased progressively among patients undergoing kidney transplant, peritoneal dialysis, and hemodialysis in that order. Hemodialysis patients were found to have an increased risk of digestive tract cancer compared with kidney transplant patients (adjusted hazard ratio = 1.9; 95% confidence interval: 1.31–2.81; *P* < 0.001). The study findings may be a useful reference for cancer-screening guidelines.

## Introduction

The incidence of cancer has been reported to be higher in patients with end-stage kidney disease (ESKD) than in the general population^1,2^. Also, as the worldwide incidence of chronic kidney disease (CKD) and cancer increases with the increase in life span^3,4^, cancer occurrence in patients with kidney disease is expected to become clinically and socioeconomically more important. Therefore, the early detection of cancer via timely screening is becoming increasingly vital^5^.

Several previous studies have compared the incidence of various cancers in ESKD patients with that in the general population^6–8^. However, the epidemiological characteristics of cancer in ESKD patients remain unclear and more national-level investigations are needed^9–11^. Thus, due to a lack of large-scale data, a cancer screening strategy for patients with ESKD has not yet been established.

Therefore, we conducted a large‐scale, retrospective cohort study using a nationwide database to investigate the cancer risk among ESKD patients, including those undergoing hemodialysis (HD), peritoneal dialysis (PD), and kidney transplant (KT). We aimed to understand characteristics of the cancer by analyzing its incidence in ESKD patients using data from the Health Insurance Review and Assessment (HIRA) service, a national registry including all patients in Korea. Using the HIRA data, we estimated the incidence of different cancers in patients with ESKD between 2008 and 2017; additionally, we examined differences in the incidence of overall and site-specific cancers between ESKD patients who underwent different treatment modalities (HD, PD, and KT).

## Materials and methods

### Data source and data collection

The HIRA service is a government organization, tasked with the assessment of medical services and maintenance of its quality standards by reviewing healthcare claims^12^. Medical providers are required to submit inpatient and outpatient claims to the HIRA for reimbursement of the cost of medical procedures covered by the Korean National Health Insurance Service (KNHIS). The KNHIS, the single public health insurance provider in Korea, has been operated by the Ministry for Health and Welfare since July 1989 and covers over 97% of the Korean population. Many previous studies using HIRA data have been published^13–15^. We extracted ESKD cohort patient data from the HIRA database including characteristics such as age; sex; diagnoses based on the International Classification of Disease, Revision 10 (ICD-10) codes; intervention procedures, and surgical procedures such as HD, PD, and KT.

This retrospective cohort study was approved by the Ajou University Hospital Institutional Review Board (IRB number: AJIRB-MED-EXP-18-499). The HIRA database was de-identified according to strict confidentiality guidelines; therefore, the requirement for informed consent was waived. Access to the HIRA database was granted by the concerned government organization (HIRA, no. M20181212478).

### Definition and selection of ESKD patients

We initially identified patients diagnosed with ESKD between January 1, 2007 and December 31, 2017 in Korea, on the basis of the presence of ICD 10-codes (N18.0–18.6, 18.9); then, we selected patients with at least one ESKD diagnosis code between 2008 and 2017 to have at least a 1-year pre-observational period prior to diagnosis. The diagnosis was also confirmed by special exemption codes specified by the Korean government as part of a national health care plan. The Korean government provides financial support to patients with rare incurable diseases such as ESKD through the KNHIS: if the diagnoses of such patients are confirmed by physicians and registered with the KNHIS, they receive a special exemption code for a reduction in co-insurance rates and medical expenses of up to 90%.

Based on the special exemption codes and treatment codes for procedures and materials associated with dialysis and transplant on their health claims, we classified the patients into three renal replacement therapy (RRT) groups: HD, PD, and KT patients. The HD group consisted of patients having a special exemption code for hemodialysis (V001) and treatment codes (O7020, O7021, O9991). The PD group consisted of patients having a special exemption code for peritoneal dialysis (V003) and treatment codes of O707, and the KT group consisted of patients having a special exemption code for kidney transplant (V005) and treatment code of R3280.

Patients aged < 18 years and those with a history of cancer either prior to the initial date of RRT or within 1 year of their inclusion in the study, were excluded. Patients who underwent < 3 months of dialysis or had no treatment codes related to RRT modalities were also excluded. When comparing cancer incidence by RRT modality, patients were excluded if they: (1) changed the dialysis modality from PD to HD; (2) changed to PD after being on HD for > 3 months; (3) changed from KT to HD or PD due to graft failure; or (4) received transplantation surgery 1 year after the first dialysis initiation (for KT patients).

### Outcomes and covariates

The primary outcome was cancer incidence by site, including digestive, urinary, respiratory, and reproductive tracts; head and neck region; and hematological, endocrine, and other organ systems. Cancer sites were defined by ICD-10 codes (C00.0–C99.9), along with in situ neoplasms (D00–D09). The lists of ICD-10 codes for specific cancer sites considered in this study are provided in Table S1. The date of initial dialysis or transplant for all patients was defined as the index date. The observation period constituted the period from the index date up until the date of the last claim or December 31, 2017, whichever was earlier. Baseline comorbidities were defined as conditions diagnosed within 1 year prior to the index date using the following ICD-10 diagnostic codes: diabetes mellitus (DM), E10-E14; hypertension, I10-I15; cardiovascular disease, I20-I25; cerebrovascular disease, I60-I69; chronic lung disease, J40-J47; and chronic liver disease, K70.3, K70.4, K72.1, K73, K74.

### Statistical analyses

To compare the baseline characteristics of the three patient groups (i.e., HD, PD, and KT), analysis of variance (ANOVA) and the chi-square test for homogeneity were used for continuous and categorical variables, respectively. The means and standard deviation of the time to cancer incidence from the index date was calculated by cancer site, which was further classified into more specific sites. Incidence rates (IR) of cancer per 10,000 person-years and their 95% confidence intervals (CIs) were estimated. The cumulative incidence of each cancer site by RRT was derived using the Kaplan–Meier curve, and log-rank tests were used to statistically compare group differences. Time was measured from the index date to the first date when the diagnosis code of cancer occurred for each site and was censored when the observation period ended. Univariate and multivariable analyses using Cox proportional hazards models were conducted to investigate the effects of baseline characteristics including age group at the time of initial dialysis or transplant, patient sex, and the presence of comorbidities such as DM, hypertension, cardiovascular disease, cerebrovascular disease, chronic lung disease, and chronic liver disease. Using KT as the reference group, unadjusted and adjusted hazard ratios (HR) and their 95% CIs were estimated. For each RRT group, IRs were stratified according to age group at the time of initial dialysis or transplant to observe any differences in cancer incidence by site among age groups. Additionally, specific cancer sites were stratified by age group and sex for each RRT type, and the three most common cancer sites were examined. All statistical analyses were based on two-sided inferences with *P* < 0.05 considered to be statistically significant, and were performed using SAS statistical software, version 9.4 (SAS Institute, Cary, NC, USA).

### Ethical approval

All procedures performed in studies involving human participants were in accordance with the ethical standards of the institutional and/or national research committees and with the 1964 Helsinki declaration and its later amendments or comparable ethical standards.

### Informed consent

For this type of study, formal consent is not required. The requirement for informed consent was waived (AJIRB-MED-EXP-18-499).

## Results

### Baseline characteristics of the study population

**Figure 1:**
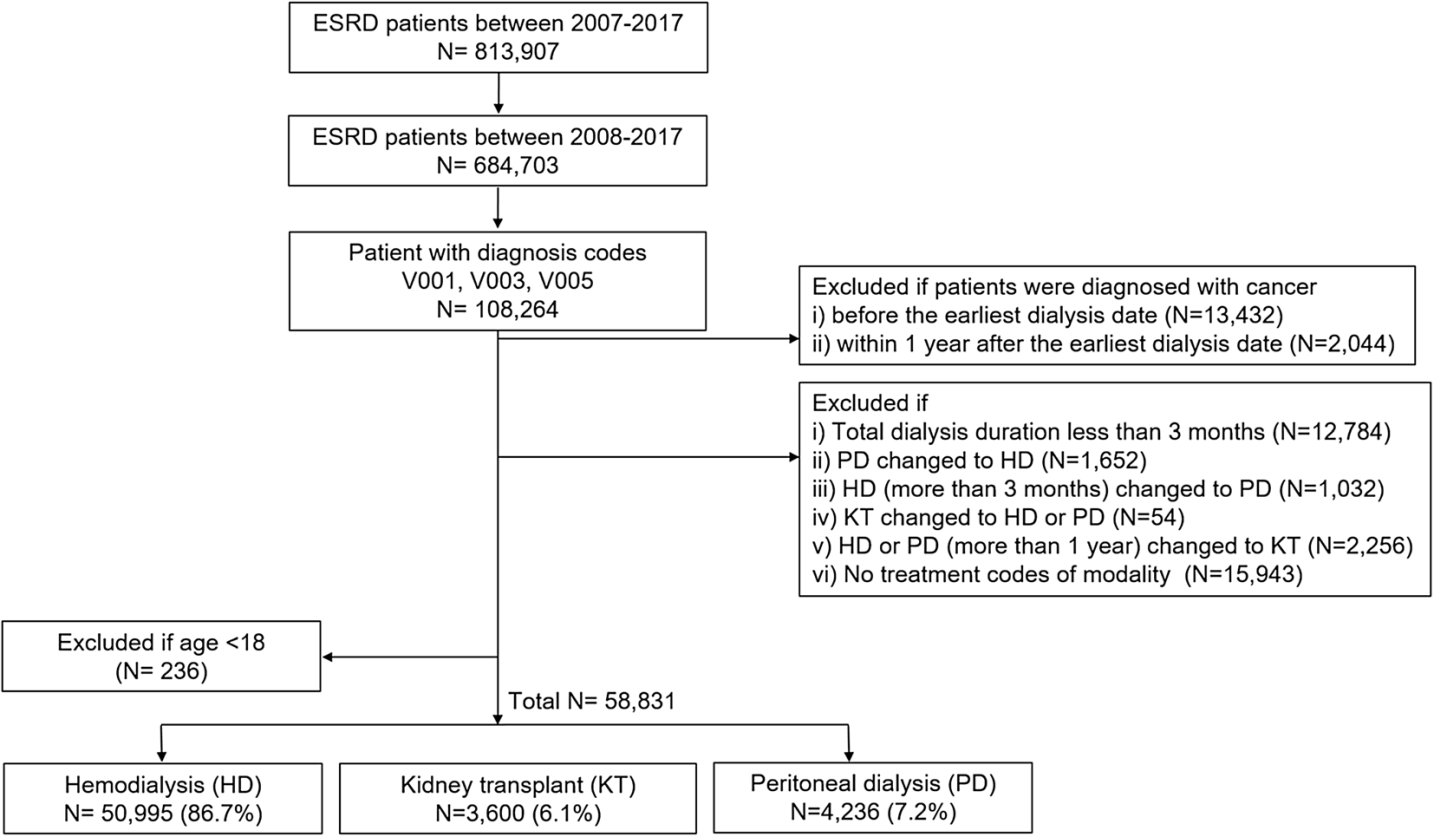
Flowchart of the study population. ESKD, End-stage kidney disease; HD, hemodialysis; PD, peritoneal dialysis; and KT, kidney transplant.

The flowchart of the study population is depicted in Fig. 1. Initially, data for a total of 813,907 patients with a diagnosis code for ESKD were extracted from the HIRA database. Then, we selected patients with at least one ESKD diagnosis code between 2008 and 2017 to have at least a 1-year pre-observational period prior to diagnosis. Among them, 108,264 patients with special exemption codes (V001, V003, V005) were obtained. Those who had cancers prior to the ESKD diagnosis (13,432) or who developed cancers within 1 year after the index date (n = 2044) were excluded. Patients who changed RRT modalities (n = 4994), who underwent < 3 months of dialysis (12,784) or who had no treatment codes related to RRT modalities (n = 15,943) were also excluded. A total of 58,831 patients met all the study inclusion criteria between January 2008 and December 2017. Of those patients, the number of HD, KT, and PD patients were 50,995 (86.7%), 3600 (6.1%), and 4236 (7.2%), respectively.

The baseline characteristics of the study population are reported in Table 1. The age of all participants on the index date was 59.5 ± 14.4 years (mean ± standard deviation [SD]), and the majority (58.5%) were male. The KT group was the youngest (age 44.5 ± 11.6 years), whereas 31.6% of HD patients were aged > 70 years. Patients were followed up for 3.4 ± 2.4 years on average. The average duration from the diagnosis of ESKD to cancer incidence was 3.3 ± 1.9 years, with no statistically significant differences among the three modalities. The mean age at the time of cancer diagnosis was 64.3 ± 12.4 years for the overall sample, whereas it was lower (51.1 ± 11.4 years) for the KT group. Comorbidities were commonly diagnosed within 1 year prior to the index date, especially in the HD group; the most prevalent being hypertension (91.5%) followed by DM (66.1%). Even in the KT group, which was the youngest, the prevalence of hypertension was 95.3%.

**Table 1:** Baseline characteristics of the study population.

Characteristics	Overall (n = 58,831)	HD (n = 50,995)	KT (n = 3600)	PD (n = 4236)	*P*-value
Age at index date, years, mean (SD)	59.5 (14.4)	61.1 (13.9)	44.5 (11.6)	53.4 (13.4)	< .0001
18–29, n (%)	1500 (2.6)	898 (1.8)	420 (11.7)	182 (4.3)	
30–39, n (%)	4054 (6.9)	2779 (5.5)	817 (22.7)	458 (10.8)	
40–49, n (%)	8974 (15.3)	6955 (13.6)	1021 (28.4)	998 (23.6)	
50–59, n (%)	13,837 (23.5)	11,636 (22.8)	1020 (28.3)	1181 (27.9)	
60–69, n (%)	13,780 (23.4)	12,592 (24.7)	301 (8.4)	887 (20.9)	
≥ 70, n (%)	16,686 (28.3)	16,135 (31.6)	21 (0.5)	530 (12.5)	
Sex, Male, n (%)	34,390 (58.5)	29,897 (58.6)	2116 (58.8)	2377 (56.1)	0.0057
Age at cancer diagnosis, years, mean (SD)*	64.3 (12.4)	65.2 (12.0)	51.1 (11.4)	58.2 (12.3)	< .0001**
Years from index date to cancer diagnosis, mean (SD)*	3.3 (1.9)	3.3 (1.9)	3.3 (1.8)	3.3 (1.9)	0.9708**
Comorbidities^¥^					
Diabetes, n (%)	38,905 (66.1)	34,651 (68.0)	1661 (46.1)	2593 (61.2)	< .0001
Hypertension, n (%)	53,825 (91.5)	46,498 (91.2)	3432 (95.3)	3895 (92.0)	< .0001
Cardiovascular, n (%)	14,750 (25.1)	13,118 (25.7)	574 (15.9)	1058 (25.0)	< .0001
Cerebrovascular, n (%)	9544 (16.2)	8929 (17.5)	197 (5.5)	418 (9.9)	< .0001
Chronic lung disease, n (%)	12,531 (21.3)	11,341 (22.2)	537 (14.9)	653 (15.4)	< .0001
Chronic liver disease, n (%)	2127 (3.6)	1925 (3.8)	67 (1.9)	135 (3.2)	< .0001
Observation duration, years, mean (SD)	3.4 (2.4)	3.4 (2.5)	3.4 (2.3)	3.5 (2.3)	< .0001

In case of patients (n = 2066/3600) who received either HD or PD before KT, mean (SD) time from HD or PD to KT is 115 (91.3) days.

SD, standard deviation; HD, hemodialysis; PD, peritoneal dialysis; KT, kidney transplant.

*Analyzed among patients with cancer (n = 3292) only.

**P-value was based on the analysis of variance (ANOVA) test; otherwise, the chi-square test.

^¥^Comorbidities were observed within 1 year before index date.

### Primary site of cancer

The primary site of cancer diagnosis is reported in Table 2. Of the 58,831 patients in the study population, 3292 patients (5.6%) were diagnosed with cancer during the follow-up period. A total of 197,199 person-years was observed. The IR per 10,000 person-years for overall cancers (cancer diagnosed at any site) was 166.9 (95% CI 161.2–172.6). The digestive tract was the most frequent site of cancer diagnosis (41.9%) and had the highest IR (68.9 per 10,000; 95% CI 65.3–72.5), which was 3.1 times higher than that in the “other organs” category (22.4 per 10,000; 95% CI 20.3–24.4). When digestive tract cancer was subdivided into more specific sites, the most commonly diagnosed cancer was colorectal cancer (31.6%), followed by liver cancer (25.2%), stomach cancer (24.1%), and pancreaticobiliary cancer (16.0%). Among the 3292 cancer cases, the most common sites of diagnosis were as follows: colorectum (n = 436 [cases]), lung (n = 417), liver (n = 348), stomach (n = 333), kidney (n = 227), pancreaticobiliary system (n = 221), skin (n = 167), prostate (n = 160), thyroid (n = 144), breast (n = 133), and bladder (n = 115).

**Table 2:** Mean time to cancer identification and primary site of malignancy among ESKD patients.

Cancer sites	Event, n (%)	Time to identification of cancer, years, mean (SD)	PY	IR per 10,000	95% CI	Specific sites	Event, n (%)
All sites	3292 (5.6)	3.29 (1.90)	197,199	166.9	161.2–172.6	–	–
Digestive tract	1380 (41.9)	3.23 (1.91)	200,284	68.9	65.3–72.5	Stomach	333 (24.1)
						Colorectal	436 (31.6)
						Liver	348 (25.2)
						Pancreaticobiliary	221 (16.0)
						Other GI tract	42 (3.1)
Urinary tract	367 (11.2)	3.46 (1.91)	201,766	18.2	16.3–20.1	Kidney	227 (61.9)
						Bladder	115 (31.3)
						Ureter	24 (6.5)
						Urethra and other	1 (0.3)
Respiratory tract	427 (13.0)	3.15 (1.77)	202,070	21.1	19.1–23.1	Lung	417 (97.7)
						Larynx, Trachea	10 (2.3)
Reproductive	283 (8.6)	3.45 (1.91)	201,965	14.0	12.4–15.6	Cervix	57 (20.1)
						Uterine	21 (7.4)
						Ovary	31 (11.0)
						Prostate	160 (56.5)
						Others	14 (5.0)
Head and neck	96 (2.9)	3.44 (1.95)	202,358	4.7	3.8–5.7	Oral cavity, lip, pharynx	55 (57.3)
						Others	41 (42.7)
Hematologic	134 (4.1)	3.15 (1.85)	202,298	6.6	55.0–77.5	Multiple myeloma	52 (38.8)
						Lymphoma	44 (32.8)
						Others	38 (28.4)
Endocrine	154 (4.7)	3.57 (2.12)	202,124	7.6	6.4–8.8	Thyroid	144 (93.5)
						Others	10 (6.5)
Other organs	451 (13.7)	3.30 (1.88)	201,675	22.4	20.3–24.4	Breast	133 (29.5)
						Skin	167 (37.0)
						Others	151 (33.5)

ESKD, end-stage kidney disease; SD, standard deviation; PY, person-years; IR, incidence rate; CI, confidence interval; GI, gastrointestinal.

### Cancer risk by renal replacement therapy modality

**Figure 2:**
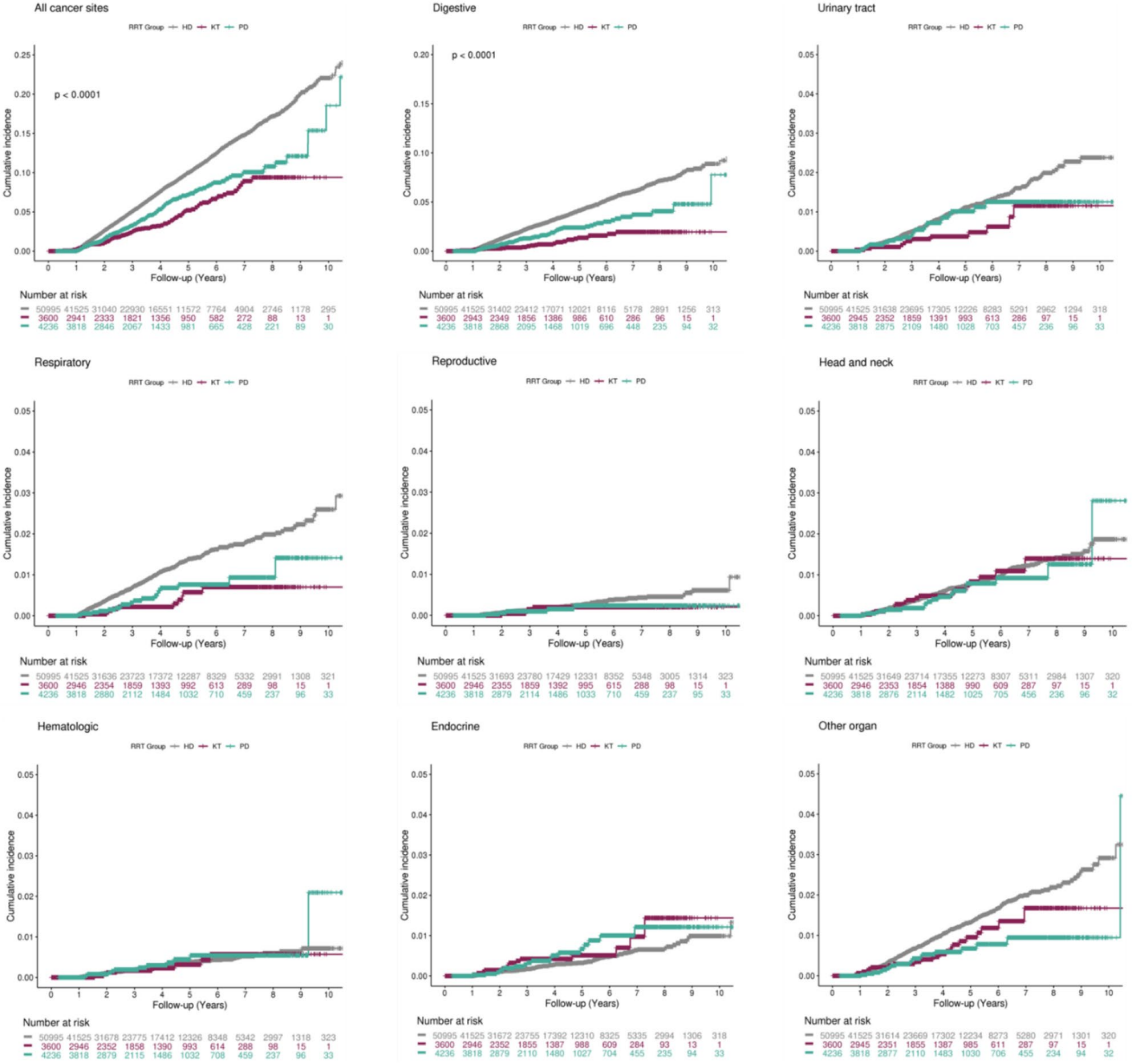
Cumulative incidence of new cancers after initial dialysis, stratified by renal replacement therapy (hemodialysis vs peritoneal dialysis vs and kidney transplant). X axis: Time since dialysis initiation; Y axis: Cumulative cancer incidence. *P*-value was based on log-rank test. HD, hemodialysis; PD, peritoneal dialysis; and KT, kidney transplant.

**Figure 3:**
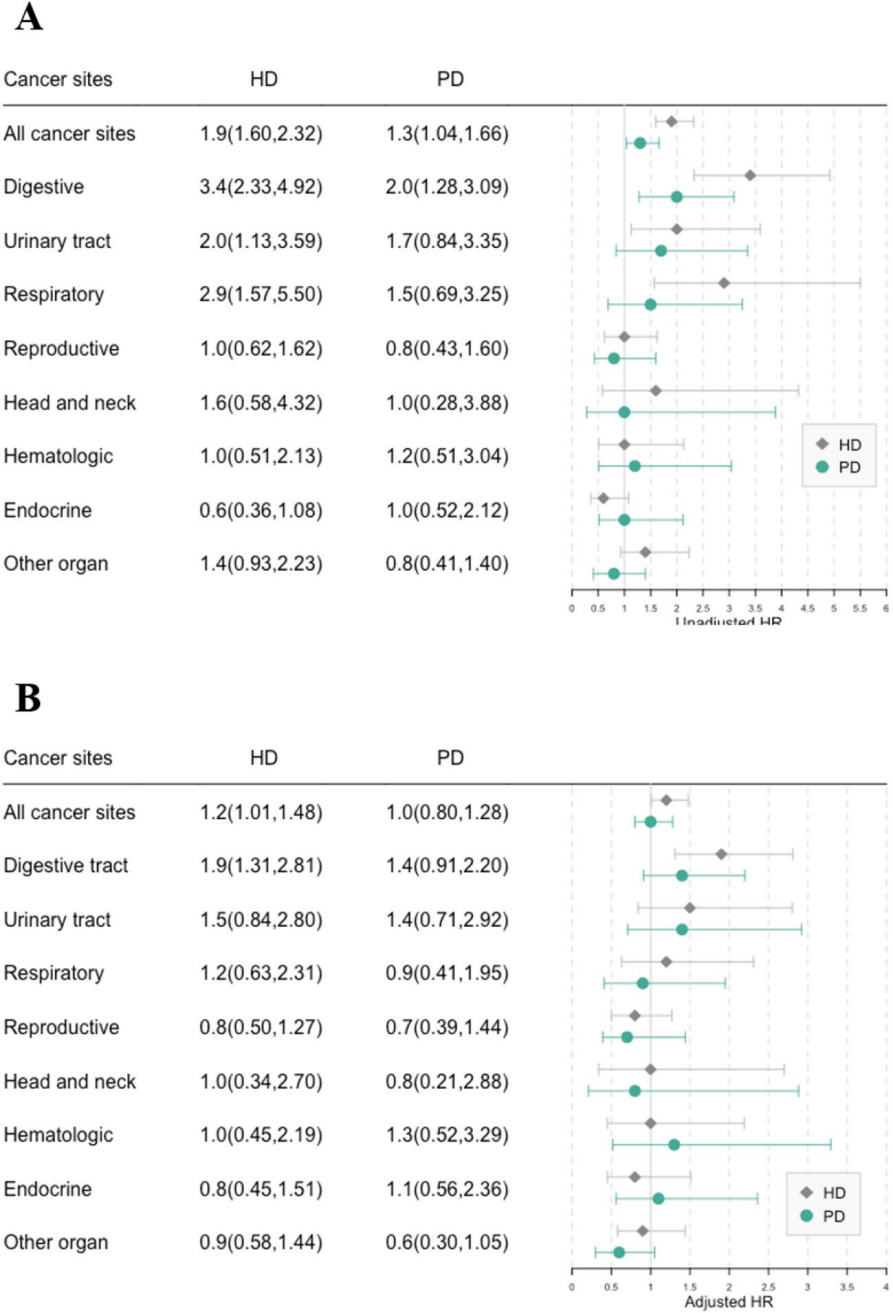
Forest plot of the cancer risk among patients undergoing hemodialysis and peritoneal dialysis versus kidney transplant. HD, hemodialysis; PD, peritoneal dialysis; HR, hazard ratio (with 95% confidence interval). (**A**) Univariate Cox regression model; (**B**) Multivariable Cox model adjusting for the effects of age group, sex, diabetes, hypertension, cardiovascular, cerebrovascular, chronic lung disease, and chronic liver disease.

The cumulative incidence of cancer at various sites from the index date, stratified by RRT, is depicted in Fig. 2. Cancer incidence for all sites progressively increased in the KT, PD, and HD groups, in that order. In addition, log-rank tests found significant differences in the incidence of digestive tract (*P* < 0.0001), respiratory tract (*P* < 0.0001), and urinary tract (*P* = 0.04) cancers between groups defined by RRT modality. The mean age at the index date, sex, and comorbidities differed between the three modality groups; therefore, we analyzed cancer risk using models that adjusted for age group, sex, diabetes, hypertension, cardiovascular, cerebrovascular, chronic lung disease, and chronic liver disease. A forest plot depicts the cancer risk in the HD and PD groups with reference to the KT group (Fig. 3). The adjusted risk of cancer was still higher in the HD group (adjusted hazard ratio [HR] = 1.2; 95% CI 1.01–1.48; *P* = 0.043) than in the KT group. When analyzed by cancer site, the risk of digestive tract cancer was significantly higher in the HD group (adjusted HR = 1.9; 95% CI 1.31–2.81; *P* < 0.001) compared with the KT group.

### Cancer risk stratified by age and sex distribution

**Figure 4:**
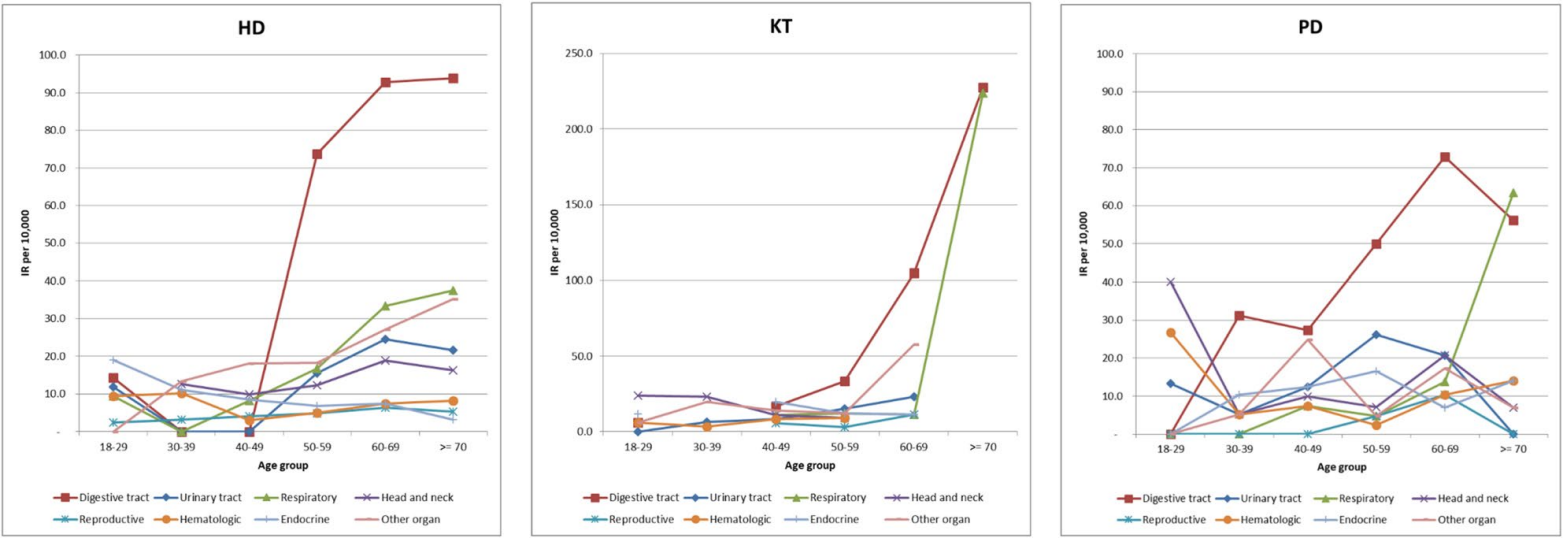
Incidence rates per 10,000 person-years by age group and renal replacement therapy for each cancer site. RRT, renal replacement therapy; HD, hemodialysis; PD, peritoneal dialysis; KT, kidney transplant; IR, incidence rate.

The IRs per 10,000 person-years by age group and RRT modality for each cancer site are reported in Table S2 and graphically depicted in Fig. 4. Over the 10-year observation period, 5.9%, 3.2%, and 4.3% of the HD, KT, and PD groups, respectively, developed cancer. In all three modality groups, it was confirmed that the incidence of all digestive tract cancers increased rapidly after 40 years of age, and that of one of the urinary tract cancers increased among participants in their 50 s and 60 s. In the HD group, endocrine and hematologic malignances were prevalent at a relatively young age (18–39 years). The KT group had the lowest IR overall; however, the risk of cancers in other organs, including breast and skin cancers, was the highest among participants in their 60 s. In the PD group, the risk of respiratory tract cancers was high among the elderly (aged ≥ 70 years), while head and neck cancers and hematologic malignancies were more prevalent among younger participants (aged 18–29 years).

Cancer incidence for the three most common sites is summarized by age group and sex to provide potentially useful information for cancer screening (Table 3 and Table S3). Among males, the incidence of lung cancer in the HD group increased starting from the 50 s age group and that of one of kidney and liver cancer was highest among the 30–49 years age group, whereas stomach cancers were common among participants in their 50 s and 60 s in both KT and PD groups. Among females, the HD group had a high incidence of thyroid, breast, and colorectal cancer in the 18–39 years, 40–59 years, and ≥ 60 years age groups, respectively. However, the most common sites of cancer were the cervix at 18–39 years for the KT group and the breast for PD group participants in their 40 s.

**Table 3:** The three most common sites of cancer incidence by age group and sex.

	RRT	Age group, year											
		18–29		30–39		40–49		50–59		60–69		≥ 70	
		Rank	Cancer site (n)	Rank	Cancer site (n)	Rank	Cancer site (n)	Rank	Cancer site (n)	Rank	Cancer site (n)	Rank	Cancer site (n)
Male	HD	1	Thyroid (5)	1	Kidney (13)	1	Liver (44)	1	Colorectral (84)	1	Lung (116)	1	Lung (111)
		2	Lung (4)	2	Liver (12)	2	Kidney (29)	2	Liver (77)	2	Colorectral (84)	2	Colorectral (74)
		3	Kidney (3)	3	Colorectral (6)	3	Stomach (28)	3	Lung (61)	3	Stomach (75)	3	Stomach (68)
	KT	1	Liver (1)	1	Other organ, others (2)	1							
			Thyroid (1)	2	Bladder (1)	2							
			Others (1)		Multiple myeloma (1)								
					Thyroid (1)								
	PD	1	Reproductive, others (1)	1	Liver (1)	1	Liver	1	Stomach (8)	1	Stomach (6)	1	Lung (7)
			Hematologic, other (1)		Ureter (1)	2	Colorectral (3)	2	Kidney (4)	2	Colorectral (5)	2	Colorectral (4)
					Multiple myeloma (1)		Kidney (3)	3	Liver (3)	3	Kidney (5)	3	Stomach (2)
					Thyroid (1)		Lung (3)				Prostate (5)		
							Thyroid (3)						
Female	HD	1	Thyroid (3)	1	Thyroid (13)	1	Breast (24)	1	Breast (32)	1	Colorectral (48)	1	Colorectral (49)
		2	Stomach (1)	2	Breast (9)	2	Thyroid (14)	2	Colorectral (22)	2	Pancreaticobiliary (1)	2	Lung (41)
			Colorectral (1)	3	Cervix (7)	3	Colorectral (13)	3	Liver (19)		Breast (26)	3	Stomach (40)
			Pancreaticobiliary (1)										
			Kidney (1)										
			Lymphoma (1)										
			Hematologic, others (1)										
	KT	1	Cervix (4)	1	Cervix (4)	1	Thyroid (7)	1	Liver (2)	1	Pancreaticobiliary (2)		
		2	Lymphoma (1)	2	Uterine (3)	2	Breast (4)	2	Thyroid (2)	2	Kidney (1)		
			Thyroid (1)	3	Breast (2)	3	Cervix (2)	3	Stomach (1)		Thyroid (1)		
									Cervix (1)		Breast (1)		
									Ovary (1)				
									Multiple myeloma (1)				
									Breast (1)				
									Skin (1)				
									Other organ, others (1)				
	PD	1	Kidney (1)	1	Liver (4)	1	Breast (6)	1	Thyroid (6)	1	Colorectral (3)	1	Lung (2)
			Cervix (1)	2	Pancreaticobiliary (1)	2	Multiple myeloma (2)	2	Kidney (5)		Liver (3)	2	Colorectral (1)
			Ovary (1)		Cervix (1)		Thyroid (2)	3	Colorectral (4)	3	Lung (2)		Pancreaticobiliary (1)
			Hematologic, others (1)		Thyroid (1)		Skin (2)				Oral cavity, lip, pharynx (2)		Multiple myeloma (1)
					Breast (1)						Thyroid (2)		Thyroid (1)
											Breast (2)		Skin (1)

RRT, renal replacement therapy; HD, hemodialysis; PD, peritoneal dialysis; KT, kidney transplant.

## Discussion

We conducted a large‐scale, retrospective cohort study using a nationwide database to investigate the cancer risk among ESKD patients, including HD, PD, and KT patients. The most common sites of cancer among ESKD patients were the colorectum, lung, and liver. After ESKD diagnosis, the average time to cancer diagnosis was about 3.3 ± 1.9 years, and there was no significant difference between the HD, PD, and KT groups. When analyzing the difference in cancer risk according to renal replacement therapy modality, the risk of digestive tract cancer in HD patients was 1.9 times that of KT patients. Therefore, our study results can serve as evidence for the recommendation that cancer screening be performed at least more than once within 3 years of the ESKD diagnosis, regardless of RRT modality, especially in younger patients or those with good performance status.

Cancer incidence in our ESKD cohort was 5.6%, similar to the 5.8% reported by Taiwanese researchers^16^ and the 5.2% reported based on Korean data^9^. There is mixed evidence on which cancers are the most prevalent among ESKD patients. Yoo et al.^11^ reported that colorectal, stomach, and kidney cancers were the most common cancers in Korean ESKD patients. Lin et al.^17^ reported that although bladder, liver, and colorectal cancers were the most common, the highest incidence ratios among ESKD patients with reference to the general population were observed for bladder, kidney, and ureter cancers. The cancer sites among ESKD patients in our study were similar to those reported in previous studies^9,11,16^. Digestive tract cancer (IR 68.9 per 10,000) was the most frequent cancer and the five most common sub-sites included the colorectum, lung, liver, stomach, and kidney. Screening for colorectal cancer using endoscopy in this clinical population is often considered to increase the risk of complications associated with the bowel cleansing preparation and with the procedure itself^18–20^. However, for ESKD patients with long life expectancies, colonoscopy for cancer screening should be considered with caution.

In a previous Korean cohort study, the CKD group demonstrated an increased risk of urinary tract malignancies compared with the general population (IR 9.4 per 10,000 vs IR 4.6 per 10,000)^10^. In the 2015 annual report of the Korean National Cancer Institute, the crude IR for urinary tract cancer was 1.68 per 10,000^21^. In our study, the IR of urinary tract cancer was 18.2 (95% CI 16.3–20.1), suggesting a higher risk of urinary tract malignancies in ESKD patients. Currently, the screening guidelines for the general population given by the Korean National Cancer Center do not include patients on dialysis and provide no information on screening for urinary malignancies^22^. Therefore, physicians should always consider the high incidence of urinary tract cancer while treating ESKD patients.

Cancer incidence was found to be higher among the younger dialysis patients than in the younger, non-dialysis cohort^9^. However, due to lack of information on cancer prevalence among younger patients, we performed the analysis stratified by age. In our study, head and neck cancer, hematologic cancer, thyroid cancer, and breast cancer were found to occur frequently among young ESKD patients aged < 40 years. In Korea, the government provides age based screening guidelines for stomach cancer (age ≥ 40 years), breast cancer (age ≥ 40 years), and colon cancer (age ≥ 50 years)^22^. Therefore, physicians should be aware of prevalent cancers (head and neck cancer, hematologic cancer, thyroid cancer, and breast cancer) when treating relatively young ESKD patients who are not eligible for cancer screening under the national program. Based on our findings, we suggest that it is necessary to proceed with cancer screening especially in young (age 18–40 years) ESKD patients who are not generally screened for cancer.

KT recipients have been shown to have a higher cancer risk than the general population^23^, and post-transplant malignancy is one of the major predictors of mortality among them^24,25^. This may be partly attributed to immunosuppression-associated malignancies in patients receiving kidney transplantation^26^. Since there is an emphasis on cancer screening related to KT in the guidelines^27^, it may be thought that cancer incidence after ESKD diagnosis may be higher in HD and PD patients than in KT patients. To exclude the incidence of cancer due to missed screening and lack of timely diagnosis in HD or PD patients, we excluded those patients who developed cancer within 1 year from the ESKD index date. In our study, digestive tract cancer was more common among HD patients than in KT patients, even after adjusting for several clinical variables. It is possible that the follow-up period was too short to detect any potential increase in the incidence of cancer due to the prolonged use of immunosuppressants.

Our population-based cohort study had some strengths. First, this was a large, nationwide study of patients based on national health insurance claims data in Korea whose registries of HD, PD, and KT patients and cancer cases are largely complete, since the Korean health insurance system covers > 97% of the Korean population. Second, KT patients as well as dialysis patients were analyzed.

Although this was a population-based cohort study that used HIRA data, the study had some limitations. First, information on the primary cause of ESKD was limited. Therefore, it was difficult to analyze the relationship between underlying kidney diseases and the IRs of various types of cancers among ESKD patients. Second, information on cancer subtypes, stages, and mortality was not available in our study. Third, there was no information on cancer-related risk factors, such as smoking status or Helicobacter pylori infection. Fourth, only incident cases were included in the analysis of differences in cancer incidence by RRT modality. Therefore, we could not analyze this association among cases that underwent a KT after several years of dialysis or among cases who had changed their dialysis modality. The mean duration of dialysis before KT in our study was only 115 days. Lastly, since our study provided only epidemiological data on cancer incidence in ESKD patients, there is an urgent need for clinical trials designed to identify ESKD patients who may benefit from cancer screening.

In conclusion, the epidemiological characteristics of cancer in ESKD patients were different for each age group. The cancer incidence rate is high within 3 years of ESKD diagnosis. Therefore, patients with good performance status and long life expectancy or young patients may be recommended cancer screening within 3 years of ESKD diagnosis.

## Supplementary Information

Supplementary Information.
