# Supplementary material for: Epidemiological characteristics of cancers in patients with end-stage kidney disease: a Korean nationwide study
Source: Sci Rep. 2021 Feb 16;11:3929. doi: 10.1038/s41598-021-83164-6 (PMC7887206; doi:10.1038/s41598-021-83164-6)
Supplement: Supplementary file 1 — Supplementary Information. [file 41598_2021_83164_MOESM1_ESM.pdf]

[SUPPLEMENTARY MATERIAL]

Epidemiological characteristics of cancers in patients with end-stage kidney disease: A Korean nationwide study

Min-Jeong Lee, MD, PhD[1†], Eunyoung Lee, MS[2,3†], Bumhee Park, PhD[2,3*], Inwhee Park, MD, PhD[1*]

[1]Department of Nephrology, Ajou University School of Medicine, Suwon, Republic of Korea

[2]Department of Biomedical Informatics, Ajou University School of Medicine, Suwon, Republic of Korea

[3]Office of Biostatistics, Medical Research Collaborating Center, Ajou Research Institute for Innovative Medicine, Ajou University Medical Center, Republic of Korea

† These authors contributed equally to this work as first authors.

Table S1. List of ICD-10 codes for specific cancer sites used in this analysis

Cancer sites	Specific sites	ICD-10 codes
Digestive tract	Stomach	C16, D00.2
	Colorectal	C18–C21, D01
	Liver	C22, D01.5
	Pancreaticobiliary	C22.1, C23–C25, D01.51–59, D01.7
	Other GI tract	C15, C17, C26, D00.1, D01.4, D01.78, D01.9
Urinary tract	Kidney	C64
	Bladder	C67, D09
	Ureter	C65, C66
	Urethra & others	C68, D09.1
Respiratory tract	Lung	C34, D02.2
	Larynx, Trachea	C32, C33, D02.1, D02.4
Reproductive	Cervix	C53, D06
	Uterine	C54, C07.0
	Ovary	C56, C07.3
	Prostate	C61, D07.5
	Others	C51, C52, C55, C57, C58, C60, C62, C63, D07.1, D07.2, D07.4, D07.6
Head & Neck	Oral cavity, lip, pharynx	C00-C14, D00.0, D02.0, D02.3
	Others	C30, C31, C69–C72, D09.2
Hematologic	Multiple myeloma	C90
	Lymphoma	C81–C86
	Others	C88, C91–C96
Endocrine	Thyroid	C73, D09.3
	Others	C37, C74, C75, C09.38
Other organs	Breast	C50, D05
	Skin	C43–C44, D03, D04
	Others	C38–C41, C45–C49, C76–C80, C97, D09.7, D09.9

Table S2. Incidence rates per 10,000 person-years by age group and renal replacement therapy for each cancer site

Cancer site	Age group	HD (n=50,995)			KT (n=3,600)			PD (n=4,236)		
		N	PY	IR per 10,000	N	PY	IR per 10,000	N	PY	IR per 10,000
All sites	-	2996	169,846	176.4	115	12,359	93.1	181	14,995	120.7
Digestive tract	18–29	6	4,211	14.2	1	1,691	5.9			-
	30–39	37	12,666	-				6	1,923	31.2
	40–49	146	29,113	-	6	3,589	16.7	11	4,016	27.4
	50–59	319	43,273	73.7	11	3,294	33.4	21	4,201	50.0
	60–69	393	42,371	92.8	9	857	105.0	21	2,881	72.9
	≥ 70	384	40,920	93.8	1	44	227.7	8	1,424	56.2
Urinary tract	18–29	5	4,213	11.9			0.0	1	753	13.3
	30–39	18	12,683	-	2	3,054	6.5	1	1,939	5.2
	40–49	46	29,289	-	3	3,591	8.4	5	4,032	12.4
	50–59	68	43,722	15.6	5	3,308	15.1	11	4,208	26.1
	60–69	105	42,792	24.5	2	868	23.0	6	2,904	20.7
	≥ 70	89	41,244	21.6						-
Respiratory tract	18–29	4	4,220	9.5						-
	30–39			-						-
	40–49	24	29,354	8.2	4	3,594	11.1	3	4,043	7.4
	50–59	73	43,770	16.7	4	3,309	12.1	2	4,225	4.7
	60–69	143	42,851	33.4	1	869	11.5	4	2,908	13.8
	≥ 70	155	41,295	37.5	1	45	223.9	9	1,421	63.4
Head and neck	18–29				4	1,686	23.7	3	750	40.0
	30–39	16	12,698	12.6	7	3,040	23.0	1	1,939	5.2
	40–49	29	29,332	9.9	4	3,595	11.1	4	4,034	9.9
	50–59	54	43,749	12.3	3	3,309	9.1	3	4,222	7.1
	60–69	81	42,833	18.9				6	2,901	20.7
	≥ 70	67	41,308	16.2				1	1,426	7.0
Reproductive	18–29	1	4,223	2.4						-
	30–39	4	12,719	3.1						-
	40–49	12	29,368	4.1	2	3,595	5.6			-
	50–59	21	43,841	4.8	1	3,310	3.0	2	4,219	4.7
	60–69	27	42,944	6.3	1	871	11.5	3	2,911	10.3
	≥ 70	22	41,397	5.3						-
Hematologic	18–29	4	4,218	9.5	1	1,690	5.9	2	749	26.7
	30–39	13	12,703	10.2	1	3,055	3.3	1	1,940	5.2
	40–49	9	29,372	3.1	3	3,591	8.4	3	4,041	7.4
	50–59	22	43,834	5.0	3	3,309	9.1	1	4,228	2.4
	60–69	32	42,932	7.5				3	2,908	10.3
	≥ 70	34	41,384	8.2				2	1,427	14.0
Endocrine	18–29	8	4,201	19.0	2	,689	11.8			-
	30–39	14	12,693	11.0				2	1,937	10.3
	40–49	25	29,331	8.5	7	3,566	19.6	5	4,031	12.4
	50–59	30	43,802	6.8	4	3,308	12.1	7	4,213	16.6
	60–69	32	2,900	7.5	1	870	11.5	2	2,907	6.9
	≥ 70	13	41,399	3.1				2	1,423	14.1
Other organs	18–29			-	1	1,690	5.9			-
	30–39	17	12,669	13.4	6	3,043	19.7	1	1,932	5.2
	40–49	53	29,292	18.1	5	3,590	13.9	10	4,027	24.8
	50–59	80	43,698	18.3	4	3,307	12.1	2	4,227	4.7
	60–69	116	42,809	27.1	5	864	57.9	5	2,901	17.2
	≥ 70	145	41,176	35.2				1	1,427	7.0

Abbreviations: HD, hemodialysis; PD, peritoneal dialysis; KT, kidney transplant; PY, person-years; IR: incidence rate

Table S3. Primary site of cancer incidence by sex

	Overall	HD	KT	PD
**Male**				
All cancers	2,145	1,977	65	103
Digestive	957(44.62)	892(45.12)	22(33.85)	43(41.75)
Urinary tract	266(12.40)	241(12.19)	9(13.85)	16(15.53)
Respiratory	343(15.99)	320(16.19)	9(13.85)	14(13.59)
Head and neck	167(7.79)	155(7.84)	2(3.08)	10(9.71)
Reproductive	69(3.22)	62(3.14)	4(6.15)	3(2.91)
Hematologic	83(3.87)	71(3.59)	6(9.23)	6(5.83)
Endocrine	62(2.89)	53(2.68)	3(4.62)	6(5.83)
Other organs	198(9.23)	183(9.26)	10(15.38)	5(4.85)
**Female**				
All cancers	1,147	1,019	50	78
Digestive	423(36.88)	393(38.57)	6(12.00)	24(30.77)
Urinary tract	101(8.81)	90(8.83)	3(6.00)	8(10.26)
Respiratory	84(7.32)	79(7.75)	1(2.00)	4(5.13)
Head and neck	116(10.11)	92(9.03)	16(32.00)	8(10.26)
Reproductive	27(2.35)	25(2.45)	0(0.00)	2(2.56)
Hematologic	51(4.45)	43(4.22)	2(4.00)	6(7.69)
Endocrine	92(8.02)	69(6.77)	11(22.00)	12(15.38)
	253(22.06)	228(22.37)	11(22.00)	14(17.95)
